# Device-Assisted Left Atrial Appendage Exclusion: From Basic Sciences to Clinical Applications

**DOI:** 10.3390/jcdd11100332

**Published:** 2024-10-18

**Authors:** Julia Izabela Karpierz, Michał Piotrowski, Krzysztof Bartuś, Radosław Chmiel, Katarzyna Wijatkowska, Artur Słomka

**Affiliations:** 1 CAROL—Cardiothoracic Anatomy Research Operative Lab, Department of Cardiovascular Surgery and Transplantology, Institute of Cardiology, Jagiellonian University Medical College, 31-008 Krakow, Poland; karpierzjuliaizabela@gmail.com (J.I.K.);; 2 Faculty of Medical Sciences in Katowice, Medical University of Silesia, 40-055 Katowice, Poland; 3 Department of Anatomy, Jagiellonian University Medical College, 31-008 Krakow, Poland; 4 Department of Cardiac Anesthesiology, Regional Specialist Hospital, 86-300 Grudziądz, Poland; 5 Department of Pathophysiology, Nicolaus Copernicus University in Toruń, Ludwik Rydygier Collegium Medicum in Bydgoszcz, 85-094 Bydgoszcz, Poland; artur.slomka@cm.umk.pl; 6 National Medical Institute of the Ministry of the Interior and Administration, 137 Wołoska Street, 02-507 Warsaw, Poland

**Keywords:** left atrial appendage occlusion, atrial fibrillation, stroke prevention, minimally invasive cardiac surgery, thromboembolism

## Abstract

Device-assisted left atrial appendage exclusion plays a crucial role in the prevention of fatal ischemic complications in patients with atrial fibrillation and contraindications to anticoagulation treatment. Various devices with different safety profiles and device-related complications are available in daily practice to perform this procedure. In this review, the anatomy, physiology, and functions of the left atrial appendage were detailed, and all available devices used for epicardial and endocardial exclusion of the left atrial appendage and their clinical outcomes were discussed. Future research should aim to further investigate the long-term effects of left atrial appendage exclusion on body homeostasis, blood coagulation, and cardiac function.

## 1. Introduction

### 1.1. Exclusion of the Left Atrial Appendage—Indications

The left atrial appendage is the most common source of thrombus in patients with atrial fibrillation. New oral anticoagulants (NOACs) are used to prevent fatal ischemic complications in patients with this most common arrhythmia. However, for certain indications, including increased risk of bleeding, such an approach is not available. In daily practice, it is recommended that patients with an increased risk of ischemic complications, as assessed by the CHAD2VASC2 score, and an increased risk of bleeding, as assessed by the HAS-BLED scale, should be treated with left atrial appendage exclusion [1].

### 1.2. Left Atrial Appendage Exclusion Techniques

Left atrial appendage exclusion techniques include surgical exclusion and/or removal and epicardial or endocardial device-assisted exclusion of the left atrial appendage via a transcatheter approach. Epicardial exclusion of the left atrial appendage is most commonly performed as an adjunctive procedure during cardiac surgery. The most commonly used device for this purpose is AtriClip (Atricure Inc., West Chester, OH, USA). Endocardial procedures for left atrial appendage occlusion include various devices inserted percutaneously into the lumen of the left atrial appendage and are usually performed as independent procedures. In addition, the LARIAT device, which combines an endovascular and an epicardial approach, should be mentioned. Each of the left atrial appendage exclusion methods has certain limitations related to the specific anatomy of the left atrial appendage and device-specific complications, which though rare, can be fatal in some cases.

### 1.3. The Aim of the Review

The aim of this review is to provide a detailed analysis of the available data on device-assisted left atrial appendage elimination, including the clinically important anatomy of the left atrial appendage, physiology, device characteristics, clinical outcomes of each device, and their impact on postoperative outcomes.

## 2. Left Atrial Appendage—Comprehensive Overview

### 2.1. Left Atrial Appendage Anatomy

The left atrial appendage is a small muscular structure with an opening in the left atrium [2,3,4,5]. It is adjacent to important anatomical structures, including the left coronary artery and its branch—the left circumflex artery, the aorta, the pulmonary trunk, the left pulmonary veins, and the mitral valve [2,3,4,5,6]. The left atrial appendage can be divided into two main parts—neck and body, also called the lobe [7]. The shape of the left atrial appendage lobe has been widely studied due to the assumption that it is associated with an increased risk of thromboembolism. However, due to the subjective nature of the first classifications of the shapes of LAA, the comparison of study results is complicated (Wang I classification) [8]. In some cases, the left atrial appendage shape was proven to be connected with an increased risk of thromboembolic events [9]. The new, simplified classification of LAA was introduced to solve this problem and provide a tool for better communication between clinicians [5,10]. The lobe of LAA is covered with pectinate muscles and is thinner than its neck, which makes it vulnerable to puncture or tearing. The neck of the left atrial appendage was defined in the past only clinically as a landing zone; however, recent research provided its detailed anatomical definition [7]. It can be divided into four walls—aortic, arterial, free, and venous, based on adjacent structures. The walls differ among themselves in terms of their length, geometry, and thickness [7]. It is especially important in left atrial appendage occlusion procedures for occlusion of the left atrial appendage, where the pressure created by the device is accompanied by fistula formation, which can end fatally [6,11,12,13]. The orifice of the left atrial appendage, the main entrance to the neck of the left atrial appendage, has various shapes, some of which are difficult to completely cover by closure devices. It has also been demonstrated that the anatomy of the left atrial appendage of the heart differs considerably between patients with atrial fibrillation and healthy controls, although the long-term effects of atrial fibrillation on the anatomy of the left atrial appendage are still being investigated [5,14]. This observation supports the previously confirmed effects of pathologies on the anatomy of the left atrial appendage and the usefulness of computed tomography in assessing the anatomy of cardiac structures [15,16]. From a clinical perspective, it should be emphasized that in some cases, the anatomical structure of the LAA, such as muscle trabeculae or the ridge of the left pulmonary veins, known as the Coumadin ridge, may misleadingly imitate the presence of thrombi in echocardiographic examinations.

### 2.2. Physiology of the Left Atrial Appendage

As previously mentioned, the fundamental mechanism underlying most strokes is the embolization of thrombotic material from the left atrial appendage [17,18,19]. Thrombus formation in this region is the result of several micro- and macroscopic factors: specific morphologies of the left atrial appendage, changes in atrial geometry, unfavorable hemodynamic conditions associated with activation of the renin–angiotensin–aldosterone system, inflammation, decrease in nitric oxide levels, and increase in the presence of growth factors [17,19]. Anticoagulation treatment with either direct oral anticoagulants or vitamin K antagonists may help counteract these unfavorable conditions and prevent thrombus formation [18,19]. It is also worth mentioning the influence of left atrial appendage occlusion on coagulation factors, since such a procedure not only mechanically prevents thrombus embolization but also has effects on the biochemistry of blood coagulation [20]. After left atrial appendage closure with the LARIAT device, a significant decrease in fibrinogen, tPA, TAFI, and PAI-1 levels was observed in patients with normal left atrial appendage. In the patients with an enlarged left atrial appendage, not only was no decrease in TAFI observed, but also a significant increase in plasminogen levels was observed [20].

### 2.3. Non-Thrombolic Role of the Left Atrial Appendage

The new descriptions of the role of LAA have been presented recently—a study by Alkhouli et al. defined the four new main roles of the left atrial appendage: influence on hemodynamics, hormonal control, atrial arrhythmia, and stem cell reservoir [21]. The HOMEOSTATIS study was the first to demonstrate the influence of left atrial appendage closure on neurohormonal regulation [22]. This role of the left atrial appendage is closely linked to changes in ANP and BNP secretion, and epicardial closure has been shown to have effects on the secretion of these natriuretic peptides. Left atrial appendage closure had a positive effect on the long-term regulation of aldosterone, epinephrine, noradrenaline, renin, and vasopressin, the levels of which were consistently and significantly lower in patients with left atrial appendage closure after the procedure [23]. The role of the left atrial appendage in hemodynamic regulation has been demonstrated in several studies. Left atrial appendage closure had a positive effect on left atrial hemodynamics in patients, although its long-term effects remain to be determined in further studies [24]. The negative influence of the left atrial appendage on stroke risk is not only related to thrombus formation, but also to the fact that approximately 27% of premature atrial contractions in patients after catheter ablation of atrial fibrillation originate from the left atrial appendage [25]. The BELIEF trial, conducted to further investigate this problem, found that extensive ablation of the left atrial appendage reduced the recurrence rate of atrial fibrillation by almost two-fold [26]. Early promising results from the use of cardiac stem cells derived from the left atrial appendage include an increase in repair capacity and a reduction in oxidative stress in cardiomyocytes [27,28]. In recent years, an additional area of concern has emerged regarding the role of the left atrial appendage—its morphology and its influence on other clinically important aspects of this structure [7].

## 3. Device-Assisted Left Atrial Appendage Exclusion Devices

### 3.1. Scope of Left Atrial Appendage Closure Devices—Epicardial or Endocardial?

**Figure 1:**
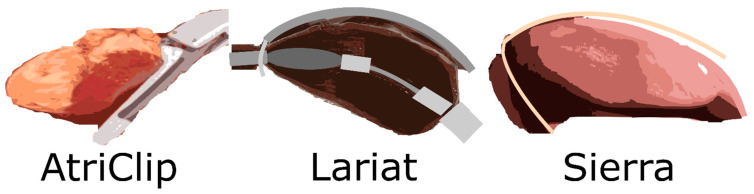
Epicardial left atrial appendage closure devices.

There are two main categories of left atrial appendage closure and occlusion procedures: endocardial and epicardial. External closure of the left atrial appendage neck closure using a loop (LARIAT), band (Sierra), or clip (AtriClip) is the basis of epicardial treatments—see Figure 1 [29,30,31].

**Figure 2:**
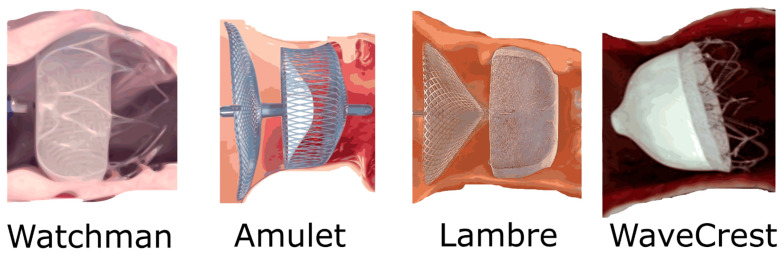
Endocardial left atrial appendage occlusion devices.

Endocardial occlusion methods are based on the percutaneous introduction of transcatheter devices that prevent embolization of the potential thrombus into the bloodstream [32,33,34,35]. There are also a few devices available in this category: WATCHMAN (the first available), AMULET, WaveCrest, and LAmbre—see Figure 2 [32,33,34,35].

Several studies have not shown the inferiority of these devices compared to treatment with direct oral anticoagulants or vitamin K antagonists, but superiority in terms of long-term non-procedural bleeding, lower rates of hemorrhagic stroke, cardiovascular deaths, and overall mortality [36]. A direct comparison of epicardial and endocardial devices has not shown superiority in the rate of cerebrovascular events or other clinically significant endpoints [32,33,37,38]. A comparison of WATCHMAN and LARIAT showed a higher number of device leaks in the endocardial device group at follow-up, but these were not related to cerebrovascular events [37]. Despite the growing interest in the left atrial appendage closure method, there are limited data comparing each method in randomized controlled trials, and most head-to-head comparisons focus on the most common devices—LARIAT and WATCHMAN [37]. The choice of the right method should be based on specific clinical-anatomical and patient-specific characteristics, which are discussed later in this review [6,31]. Each device is also associated with a specific set of device-related complications that could be avoided if they were known in advance [6,31]. Both epicardial and endocardial methods of left atrial appendage closure have lower mortality than surgical closure of the left atrial appendage or other cardiothoracic surgeries [39,40,41,42]. Detailed indications, contraindications, and main factors impacting the choice of the device are presented in Table 1.

### 3.2. Endocardial Left Atrial Appendage Closure Devices

As mentioned above, one of the options for left atrial appendage closure is the use of certain transcatheter devices [32,33,34,43]. The catheter is introduced through the femoral approach and then the left atrial appendage area is reached by penetrating the atrial septum [44]. The occluder is inserted into the neck of the left atrial appendage in the so-called “landing zone” to close the possible route for embolization of the thrombus into the bloodstream [7,44]. In this section, available devices for endocardial closure of the left atrial appendage are discussed.

#### 3.2.1. WATCHMAN and WATCHMAN FLX

The first introduced and most commonly used endocardial closure device for the left atrial appendage is WATCHMAN and its next generation: WATCHMAN FLX (Boston Scientific, Marlborough, MA, USA). It has been studied in several trials (PREVAIL, CAP2, PINNACLE FLX) and showed promising effects in nearly 100% of patients with complete left atrial appendage occlusion after one year of follow-up [45,46,47]. Warfarin discontinuation rates were 92% and 93%, respectively, and direct oral anticoagulant discontinuation rates were 96.2% [45,46,47]. The device is available in sizes ranging from 20 mm to 35 mm. The device consists of a nitinol frame, fabric, a threaded insert in the center, and barbs for fixation [48]. The details of the procedure have been described by Kar et al [45]. The procedure for implanting the WATCHMAN device is performed under general anesthesia. Transesophageal echocardiography and angiography are used to select the device size and to guide during the procedure. Femoral venous access is used for transseptal puncture, and a guidewire and pigtail are used to guide and position the sheath. The available size of the delivery system is 14 Fr. The device is inserted into the left atrial appendage by forming a “ball”. It can then be fully deployed by either moving the device distally out of the sheath or leaving the ball in place, or by combining both methods. To activate the fixation hooks and adjust the device to the ear, the operator had to apply forward pressure on the delivery cable for at least 10 s after device insertion. If the position was not ideal, the “ball” could be used to partially or fully retrieve the device and reposition it both proximally and distally.

#### 3.2.2. Amplatzer Amulet

The Amplatzer Amulet left atrial appendage closure device (Abbott, Abbott Park, IL, USA) is similar to the WATCHMAN device projected to be used as a percutaneous device; however, the main difference should be noted, including the fixing technique (anchors or double-lined anchors in WATCHMAN and hooks in Amulet) and presence of orifice closing disk, crucial for additional leakage prevention, which is exclusively observed in Amulet only [49,50]. A direct comparison between these two devices showed the superiority of Amulet in terms of post-procedure leaks and inferiority in terms of total complications [51]. In addition, there were no differences in significant clinical endpoints, such as stroke, major post-procedure bleeding, and death from any cause [51]. Amplatzer Amulet is supplied with disk sizes ranging from 22 mm to 41 mm. The delivery systems range in size from 14.4 Fr to 16.5 Fr. The main part of the device consists of a flap with stabilizing hooks and a fixed cover disk. The procedure is similar to that described in the case of WATCHMAN [49,51].

#### 3.2.3. LAmbre

The LAmbre atrial appendage (Lifetech Scientific Corp., Shenzhen, China) consists of a delivery system (10.4–12.3 Fr sheath), a hooked umbrella, and a cover that can be adjusted according to the size. The eight small hooks on the distal periphery of the umbrella and the eight U-shaped ends serve as a double stabilization by engaging the walls of the left atrial appendage and its trabeculae to ensure occlusion of the structure. The cover and the umbrella are connected by a central waist that allows the cover to align according to the left atrial wall. A clinical trial evaluating LAmbre in the United States was approved by the FDA after a trial conducted in China provided data on the high efficacy and safety of the device; similar studies are also being conducted in Europe [52,53,54]. There have been no randomized controlled trials comparing LAmbre with other devices, although cohort studies suggest similar outcomes for LAmbre, WATCHMAN, and Amplatzer [48,55,56,57].

#### 3.2.4. Wavecrest

Wavecrest (Biosense Webster, Irvine, CA, USA) is a single-lobe device with a nitinol frame. It also features a polytetrafluoroethylene layer facing the left atrium and a foam layer facing the left atrial appendage. It is fixed in the left atrial appendage by 10 bidirectional anchors and 10 single anchors [43,44]. In 2014, the results of the WAVECREST 1 trial were presented. In Europe, Australia, and New Zealand, the WaveCrest device was used in 73 patients with non-valvular atrial fibrillation. In 93% of cases, the left atrial appendage was closed successfully and with few complications. There were two cases of acute cardiac tamponade but no device-related strokes [58]. The WAVECREST II trial was conducted to compare the Wavecrest device with WATCHMAN and investigate whether it met the same safety and efficacy standards. Nevertheless, the trial was stopped due to “device design changes” and no results were published, although 248 patients participated in the trial [59].

### 3.3. Epicardial Left Atrial Appendage Closure Devices

Epicardial left atrial appendage closure is a minimally invasive hybrid procedure that involves multiple catheters and techniques to close the left atrial appendage from the outside, either with a loop, band, or clip. They require epicardial access to the left atrial appendage and are therefore most often performed during other cardiothoracic procedures [1,31]. The detailed data on each of the devices are discussed in this section.

#### 3.3.1. LARIAT

The LARIAT system involves a hybrid approach, including the endocardial use of three catheters and the epicardial LARIAT suture system. One is inserted into the femoral vein to introduce a 0.025-inch magnetic-tipped endocardial guidewire into the left atrium. Second, a 0.035-inch magnetic-tipped guidewire is inserted into the epicardium, punctured in the pericardium, and placed in the left atrial appendage on the outside of the left atrium [21,60,61,62]. Precise connection is enabled by the opposite polarity magnet on each wire [22,60,61,62]. Third, a 15 mm compliant occlusion balloon catheter is used to locate the left atrial appendage and enable highly precise occlusion of the left atrial appendage [21,60,61,62]. Although the FDA and physicians raised concerns about the safety of the LARIAT device as early as 2015 due to a significant number of severe complications reported in a national study by Lakkireddy et al., the introduction of new pericardial access methods in response to the reported problems has increased the safety of epicardial procedures to a similar level to endocardial procedures [63,64,65]. Despite similar safety, implantation of the LARIAT device is still a more invasive and stressful procedure for the patient—it requires pericardial drainage and intensive care monitoring after the procedure [63]. Another fact is worth mentioning: a recent multicenter registry showed significantly lower systolic blood pressure after 3 and 12 months in patients treated with epicardial systems [66]. This interesting fact still needs to be investigated in preferably blinded studies to prove or refute the results of the mentioned registry. If confirmed, the reduction in the number of antihypertensive medications required after the use of an epicardial system would be an important factor in the indications for either epicardial or endocardial left atrial appendage closure methods [66].

#### 3.3.2. AtriClip

AtriClip (AtriClip PRO Device, AtriCure, Dayton, OH, USA) is a fully thoracoscopic device for complete closure of the left atrial appendage [31,67]. The AtriClip device consists of a disposable holder with a head that can be moved laterally by 60 degrees and up/down and an automatically closing clip positioned in a deployment loop. The patient is placed in the supine position and intubated with a double-lumen intratracheal tube under general anesthesia. A transesophageal echocardiography probe is inserted to visualize the patient’s heart, and ventilation of the right lung is started thereafter. Three thoracoscopic ports are then inserted: two through the third and sixth intercostal spaces in the mid-axillary line into the left pleura (working ports) and one through the fourth intercostal space in the anterior axillary line (for the thoracoscopic camera). The working area is created by CO_2_ inflation. A pericardiectomy parallel to the phrenic nerve is performed to visualize the left atrial appendage. To obtain better access to the left atrial appendage, stay sutures are placed near the lower edge of the pericardium. The incision in the sixth intercostal space, expanded to 2–3 cm, is used to install the AtriClip device. Under echocardiography control, the device is attached to the base of the left atrial appendage. Special care is taken to avoid a stump. If echocardiography shows that the left atrial appendage is partially occluded or there is a remnant of the left atrial appendage after surgery, the position of the clip can be adjusted by reopening it before fully deploying it. Finally, complete exclusion of the left atrial appendage is verified by echocardiography [31,67,68].

#### 3.3.3. Sierra

The Sierra Ligation System (Aegis Medical Innovations, Vancouver, BC, Canada) is similar to LARIAT, but has some important differences: First, the classic loop is converted into a band that is placed around the left atrial appendage to exclude it [69]. The band is then lashed with a lock to secure the position of the device. It is also worth mentioning that, unlike LARIAT, Sierra does not require a transseptal puncture and is inserted as a single access through the subxiphoid area. It is available in one size and is used in cases of unsuitable anatomy for endocardial devices. The LASSO-AF clinical trial is being conducted to investigate the safety and efficacy of this device. However, only seven participants were enrolled and, to date, no results have been published [70].

## 4. Device Related Complications and Its Prevention

Describing the left atrial appendage exclusion complications, it should be noted that it should be divided into two main types: access-related complications and device-related complications. For endocardial devices and LARIAT devices, access-related complications include bleeding in the access region and complications associated with a transseptal puncture, including the left atrium, right atrium, right ventricle, or aorta perforation [31,37,38]. For AtriClip, such complications include all complications associated with sternotomy or minimally invasive thoracotomy, as it is impossible to differentiate between the impact of the left atrial appendage exclusion and concomitant procedures on its occurrence [37,38]. For Sierra devices, leakage, bleeding, or pericardial effusion are the most common access-related complications. Device-related complications for all devices include cardiac tissue (especially left atrial appendage) tear, laceration, or puncture—in some of the cases, such complications require surgical intervention [31,37,38]. For endocardial devices, device embolization is a very dangerous complication, which may lead to device localization inside the left ventricle, left atrium, or even aorta. In some cases, it can be treated percutaneously; however, in the worst event, surgical intervention is needed. Additionally, device embolization negates the impact of the procedure on stroke prevention and may lead to stroke in the patients. In some cases, there can be thrombus formation observed in the surroundings of the device, which may be the source of thromboembolic cerebral or mesenteric ischemia. For epicardial devices, especially AtriClip, there were cases of left circumflex artery occlusion described [6]. Additionally, the clip can damage adjacent tissue, which may require surgical intervention. Exclusively for WATCHMAN, there were cases of left atrial appendage to left circumflex artery fistula formation, which was associated with constant pressure to the left atrial appendage walls [10,11,12]. The pericardial effusion is one of the most common complications for both epicardial and endocardial left atrial appendage closure. In most cases, the source of the effusion is unknown. It should be noted that the pericardial effusion may be present as an effect of tissue laceration or perforation and should be examined and treated with caution [31].

There are several ways to improve surgical performance and avoid some of these complications. It should be known, however, that the left atrial appendage exclusion is safe, even when combined with concomitant procedures and surgical ablation [71,72,73].

The anatomy of cardiac structures remains largely unexplored, although recent important clinical findings have improved the safety of patient-specific cardiac surgeries and procedures [74]. The choice of a left atrial appendage closure device or the method used to eliminate the left atrial appendage should be made with great knowledge of the anatomy of the neck of the left atrial appendage to reduce the possibility of problems and leaks near the device [7]. The spatial relationships between the left atrial appendage and surrounding structures should also be evaluated. One of the rare, albeit fatal, complications of left atrial appendage closure with epicardial devices related to the anatomy of the region is occlusion of the left circumflex artery (Cx) [6,75]. Cx is located in close proximity to the left atrial appendage and can be damaged during the procedure. This problem can be largely avoided if the surgeon uses his clinical experience and appropriate preoperative visualization. There are several ways to study the patient’s unique spatial relationships in this region, including the recently expanded 3D visualization methods based on radiological images [6,76]. Analysis of spatial relationships using the aforementioned methods could be helpful in preoperative localization of potential danger zones to further reduce the likelihood of iatrogenic complications and improve treatment outcomes.

Another potential option to reduce the risk of complications during endocardial device procedures is fusion imaging, which is achieved by real-time synchronization of transesophageal echocardiography and fluoroscopic C-arm images [77,78]. An X-ray image and up to three echocardiographic views can be displayed simultaneously thanks to the result of the co-registration procedure. This option could be beneficial for experienced surgeons to further increase the safety of the procedure.

## 5. Future Directions in Research

Future research should focus on further evaluation of differences in biochemical and mechanical outcomes between endocardial and epicardial left atrial appendage exclusion procedures. Additionally, it should be considered to further expand national registries, to include the type of device used in left atrial appendage exclusion, which is everyday practice for aortic valve replacement. Additionally, some of the markers of cardiac arrest can be considered to be implemented in research regarding the impact of the left atrial appendage exclusion on cardiac function [79].

## 6. Conclusions

The left atrial appendage exclusion plays not only an important role in stroke prevention in patients with atrial fibrillation, but also has a great systemic impact on coagulation and hormonal homeostasis. A wide range of devices for left atrial appendage exclusion was developed, with broad evidence supporting its safety and usefulness.

## Figures and Tables

**Table 1:** Epicardial and endocardial devices, indications, contraindications, additional benefits, and limitations. LAA—left atrial appendage.

Device Group	Epicardial Devices			Endocardial Devices			
Device Name	LARIAT	AtriClip	Sierra	WATCHMAN	Amulet	LAmbre	WaveCrest
Indications	Patients qualified for isolated LAA closure	Patients qualified for isolated LAA closure or as concomitant procedure during cardiac surgery	Patients qualified for isolated LAA closure	Patients qualified for isolated LAA closure	Patients qualified for isolated LAA closure	Patients qualified for isolated LAA closure	Patients qualified for isolated LAA closure
contraindications	Unfavorable LAA anatomy; Previous cardiosurgical procedures; Vascular access unavailable	Too small LAA;	Unfavorable LAA anatomy; Previous cardiosurgical procedures	Vascular access unavailable; multiple lobe LAA; LAA size	Vascular access unavailable; Atypical LAA orifice shape or size	Vascular access unavailable; Atypical LAA orifice shape or size	Vascular access unavailable; multiple lobe LAA; LAA size
Additional benefits	Lack of foreign body inside patient’s heart	Lack of foreign body inside patient’s heart	Lack of foreign body inside patient’s heart, does not require endocardial access	Does not require epicardial access	Additional closure of LAA orifice with the disc	Additional closure of LAA orifice with the disc	Does not require epicardial access
Additional limitations	Requires both epicardial and endocardial access	Clip located in epicardium, if slips may lead to tears and damage adjacent tissue	Highly dependent on LAA anatomy—no guidewire inside LAA.	Foreign body inside patient’s heart, may embolize, may leak, the closure may not be complete	Foreign body inside patient’s heart, may embolize, may leak	Foreign body inside patient’s heart, may embolize, may leak	Foreign body inside patient’s heart, may embolize, may leak, the closure may not be complete

## Data Availability

No new data were created or analyzed in this study. Data sharing is not applicable to this article.
